# Serine Metabolism Tunes Immune Responses To Promote *Oreochromis niloticus* Survival upon *Edwardsiella tarda* Infection

**DOI:** 10.1128/mSystems.00426-21

**Published:** 2021-08-24

**Authors:** Dai-Xiao Yang, Man-Jun Yang, Yue Yin, Tian-Shun Kou, Liao-Tian Peng, Zhuang-Gui Chen, Jun Zheng, Bo Peng

**Affiliations:** a Center for Proteomics and Metabolomics, State Key Laboratory of Biocontrol, Guangdong Key Laboratory of Pharmaceutical Functional Genes, School of Life Sciences, Southern Marine Science and Engineering Guangdong Laboratory (Zhuhai), Sun Yat-sen University, Guangzhou, People’s Republic of China; b Laboratory for Marine Biology and Biotechnology, Qingdao National Laboratory for Marine Science and Technology, Qingdao, China; c Department of Pediatrics, The Third Affiliated Hospital of Sun Yat-sen University, Guangzhou, China; d Faculty of Health Sciences, University of Macau, Macau SAR, China; Georgia Institute of Technology

**Keywords:** glutathione, metabolomics, overactive immune response, reactive oxygen species, serine

## Abstract

Overactive immune response is a critical factor triggering host death upon bacterial infection. However, the mechanism behind the regulation of excessive immune responses is still largely unknown, and the corresponding control and preventive measures are still to be explored. In this study, we find that Nile tilapia, *Oreochromis niloticus*, that died from Edwardsiella tarda infection had higher levels of immune responses than those that survived. Such immune responses are strongly associated with metabolism that was altered at 6 h postinfection. By gas chromatography-mass spectrometry-based metabolome profiling, we identify glycine, serine, and threonine metabolism as the top three of the most impacted pathways, which were not properly activated in the fish that died. Serine is one of the crucial biomarkers. Exogenous serine can promote *O. niloticus* survival both as a prophylactic and therapeutic upon *E. tarda* infection. Our further analysis revealed exogenous serine flux into the glycine, serine, and threonine metabolism and, more importantly, the glutathione metabolism via glycine. The increased glutathione synthesis could downregulate reactive oxygen species. Therefore, these data together suggest that metabolic modulation of immune responses is a potential preventive strategy to control overactive immune responses.

**IMPORTANCE** Bacterial virulence factors are not the only factors responsible for host death. Overactive immune responses, such as cytokine storm, contribute to tissue injury that results in organ failure and ultimately the death of the host. Despite the recent development of anti-inflammation strategies, the way to tune immune responses to an appropriate level is still lacking. We propose that metabolic modulation is a promising approach in tuning immune responses. We find that the metabolomic shift at as early as 6 h postinfection can be predictive of the consequences of infection. Serine is a crucial biomarker whose administration can promote host survival upon bacterial infection either in a prophylactic or therapeutic way. Further analysis demonstrated that exogenous serine promotes the synthesis of glutathione, which downregulates reactive oxygen species to dampen immune responses. Our study exemplifies that the metabolite(s) is a potential therapeutic reagent for overactive immune response during bacterial infection.

## INTRODUCTION

Bacterial infections, especially those by multidrug-resistant bacteria, are a globally challenging issue that threatens both sustainable farming and human health (1, 2). Although antibiotics are still the primary choice to treat bacterial infection, the exhausted availability of effective antibiotic reservoirs is challenging (3). Meanwhile, only a few new antibiotics have been successfully developed in the last 20 years, which is largely unable to meet the demand (4). This situation is worsening in the context of animal farming. Antibiotics have been extensively used in animal industry for the purpose of both growth promotion and disease prevention since the 1960s (5). This practice results in the wide distribution of antibiotics residuals in the environment as well as the emergence and wide spread of resistant genes around the world (6). Many countries have employed policies against the use of antibiotics as feed additives in animal farming. However, this requires novel approaches to replace the use of antibiotics to ensure productivity and health (7, 8). The most notable approach is vaccination, which has proved to be effective against infectious diseases in both humans and animals (9, 10). However, the technical challenge is the limited understanding of immune systems of aquatic animals as well as the presence of diverse pathogens in the habitat or breeding environment, which makes the vaccines effective only in certain species of animals and against specific types of bacterial strains (11–13). The exogenous substances as immunostimulants to boost host immune systems is another option to control infectious diseases (14). Although immunostimulants, such as herbal extracts, have been widely used in aquaculture to boost the immune system of fish, they also possess certain disadvantages, such as high cost or limited effectiveness upon parenteral administration (15). Therefore, alternative strategies are urgently required.

Reprogramming metabolomics is a recently proposed strategy to modulate host immune systems to combat bacterial infection (16). This strategy takes advantage of the functional metabolomics that allow the identification of crucial biomarkers. The identified biomarker can be used to reprogram the host metabolome to modulate its immune response. More importantly, it is well recognized that metabolites are key mediators of immune responses (14, 17). The identified biomarkers, such as glucose, stearic acid, palmitate, malate, and aspartic acid, can reprogram host metabolomes to enhance their innate immune responses to bacterial infection (18–21). By comparing the metabolomes of the dying host with those of the surviving host upon bacterial infection at the 50% lethal dose (LD_50_), crucial biomarkers can be obtained through the analysis of differences in metabolomic profiling. Exogenous supplementation of these metabolites provided to the host prior to the infection can promote host survival. In addition to exerting proinflammatory effects, metabolites can also exert anti-inflammatory function. Tryptophan was recently shown to increase NADPH production that attenuates immune responses (14). Additionally, antibiotic-resistant bacteria can persist in the host longer than the antibiotic-sensitive strains, which is a potential hazard to be spread across different species. Maltose and phenylalanine can potentiate the host to clear ceftazidime-resistant and levofloxacin-resistant Vibrio alginolyticus (17, 22). This strategy has proved to be effective against bacterial infection even if the host was under unfavorable conditions, like high temperature, the etiological cause of disease outbreak in aquaculture (23). Taken together, these studies suggest that metabolites are the active players in immune responses, and modulating the host immune system through metabolism may be a promising anti-infection strategy in animal farming that is low toxicity and eco-friendly.

In searching for efficacious metabolites to combat bacterial infection, we adopt the Edwardsiella tarda*-Oreochromis niloticus* infection model. *E. tarda* is a versatile pathogen that can infect a broad range of hosts, from fish to human, and causes huge economic loss in aquaculture (24). *O. niloticus* is a fish model for the study of immunity of finfish. More importantly, *O. niloticus* is raised throughout the world for economic purpose (25). *E. tarda* is frequently isolated from cultured tilapia, representing a typical pathogen for the tilapia industry (26). By functional metabolomics, we found that *O. niloticus* exhibits two distinct metabolomes as early as 6 h postinfection by the LD_50_ of *E. tarda*. Interestingly, one of the two metabolomes clustered with that of live fish surviving at 5 days postinfection. These two metabolomes share a crucial biomarker, serine. We further demonstrate that exogenous serine promotes *O. niloticus* survival against *E. tarda* infection through reducing reactive oxygen species (ROS) production.

## RESULTS

### Differential immune responses of *Oreochromis niloticus* are associated with consequences of *E. tarda* infection.

**FIG 1:**
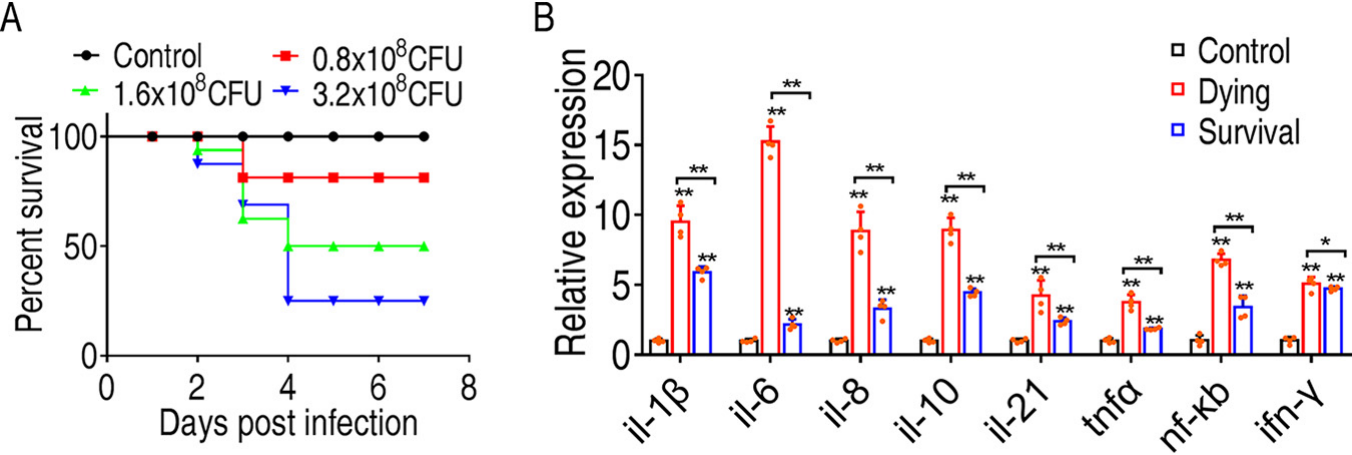
Differential immune response of *O. niloticus* is associated with the consequence of *E. tarda* infection. (A) Determination of the lethal dose of *E. tarda* to *O. niloticus. O. niloticus* fish were infected with either 0.8 × 10^8^, 1.6 × 10^8^, or 3.2 × 10^8^ CFU/fish. Cumulative fish death was monitored for a total of 14 days. (B) qRT-PCR to quantify cytokine expression in head kidneys of control, dying, or surviving fish 48 h postinfection. All of the statistical analyses were performed with Mann-Whitney U test unless otherwise indicated. *, *P < *0.05; **, *P < *0.01. Error bars represent means ± standard errors of the means (SEM) from at least three biological replicates. All of the experiments were repeated at least three times.

To develop measures to control infection by *E. tarda*, we infected *O. niloticus* with *E. tarda* at three different doses and identified 1.6 × 10^7^ as the LD_50_ of *E. tarda* (the dose that causes 50% *O. niloticus* death) (Fig. 1A). This dose was chosen for the subsequent study. Under this dose, dying fish and surviving fish at 48 h postinfection were collected, and the head kidneys, one of the major immune organs in finfish, were removed to quantify the expression of immune-related genes, including *il-1β*, *il-6*, *il-8*, *il-10*, *il-21*, *tnfα*, *nf-κb*, and *ifn-γ*. Our results showed that *E. tarda* infection induced much higher cytokine expression than that of the control group. Interestingly, dying fish had higher cytokine expression than surviving fish (Fig. 1B). This result indicates that excessive immune responses are a profound factor in host death.

### Metabolic shift upon *E. tarda* infection is associated with fish survival.

**FIG 2:**
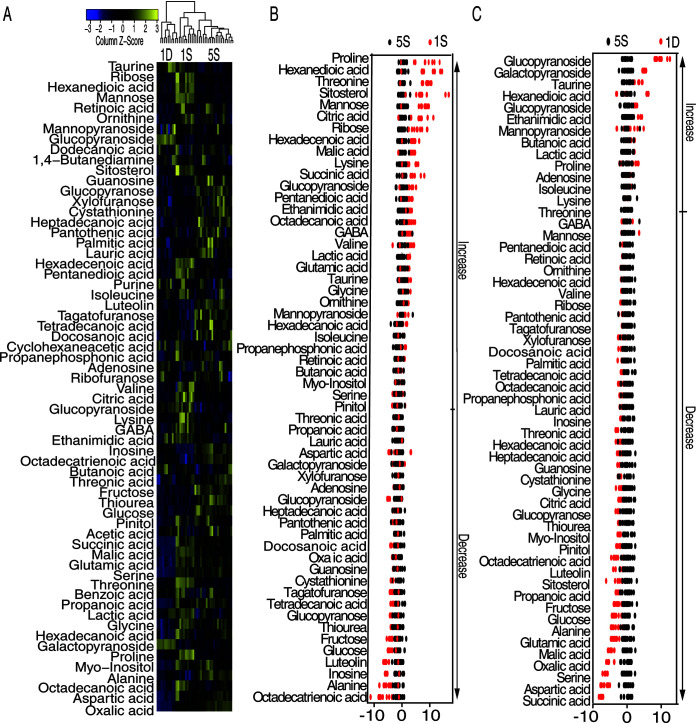
Metabolic shift upon *E. tarda* infection. (A) Heat map showing relative abundance of metabolites (Wilcoxon *P* < 0.01) in 5S, 1D, and 1S groups. Scale is shown at bottom, where blue to yellow represents low to high abundance. (B) Z-score plot of differential metabolites based on 5S. Z-score varied between −5.82 and 10.64 for 1S to 5S. Each point represents one metabolite in one technical repeat and is colored by sample types. (C) Z-score plot of differential metabolites based on 5S. Z-score varied between −7.85 and 9.71 for 1D to 5S. Each point represents one metabolite in one technical repeat and is colored by sample type.

Metabolism has been suggested to be an active player that guides immune responses in immune cells (27). Based on our previous reports that metabolic state was altered in the host during bacterial infection and can be harnessed for modulating immune response, we hypothesize that metabolism is altered in the symptoms of infection, from which we could identify a metabolite(s) to tune immune responses for fish survival. We designed our experiment as summarized in Fig. S1 in the supplemental material. Briefly, we randomly divided the fish into two groups. Fish in group 1 (*n* = 10) were challenged with the LD_50_ of *E. tarda* for 6 h and then sacrificed to collect the monocytes/macrophages from head kidneys for metabolomic analysis, whereas the fish of group 2 were challenged in the same way as group 1. However, only surviving fish at day 5 postinfection were collected. Our results demonstrated that fish would not die after day 5 postinfection. The monocytes/macrophages of surviving fish were collected for metabolomic analysis. The isolated cells were devoid of any contamination of bacteria by plating. A total of 20 samples (with 10 samples from each group) were analyzed by gas chromatography-mass spectrometry (GC-MS), and each sample had two technical replicates. In total, 40 data points were generated. Pearson correlation coefficients were obtained to confirm the reproducibility of the data of 5S (Fig. S1B). The biological replicates showed lower variation, as demonstrated by relative standard deviation (RSD), the mean of which was 2.59% ± 0.04%, ensuring that the variability of the abundance of metabolites was attributed to the biological differences but not due to methodology. Lastly, 253 peaks were aligned and the metabolites identified by searching against National Institute of Standards and Technology (NIST) and validated by their retention indices (RIs) (Data Set S1). The internal standard, ribitol, and any other metabolite corresponding to the solvents were excluded following multivariate analysis. A total of 63 metabolites were identified (Data Set S2). Among the metabolites that were searched against MetaboAnalyst 5.0, 21 metabolites were carbohydrates (33%), 17 metabolites were amino acids (27%), 10 metabolites were lipids (16%), 5 metabolites were nucleotides (8%), and 10 metabolites were others (16%) (Fig. S1C). All of the metabolites were clustered together and displayed as a heat map in Fig. 2A.

10.1128/mSystems.00426-21.1 FIG S1 Metabolomic analysis of 1S, 1D, and 5S tilapia upon *E. tarda* infection. (A) Schematic representation of experiment workflow. (B) Reproducibility of the data of 5S. (C) Functional categories of the identified metabolites. (D) Heat map showing relative abundance of difference metabolites in 5S, 1D, and 1S groups. Heat map scale (blue to yellow: low to high abundance) is shown at bottom. Download FIG S1, TIF file, 1.2 MB. Copyright © 2021 Yang et al. 2021 Yang et al. https://creativecommons.org/licenses/by/4.0/ This content is distributed under the terms of the Creative Commons Attribution 4.0 International license.

10.1128/mSystems.00426-21.6 DATA SET S1 Lists of identified metabolites. Download Data Set S1, XLSX file, 0.02 MB. Copyright © 2021 Yang et al. 2021 Yang et al. https://creativecommons.org/licenses/by/4.0/ This content is distributed under the terms of the Creative Commons Attribution 4.0 International license.

10.1128/mSystems.00426-21.7 DATA SET S2 Statistical analysis of metabolomic data of 1S, 1D, and 5S. Download Data Set S2, XLSX file, 0.04 MB. Copyright © 2021 Yang et al. 2021 Yang et al. https://creativecommons.org/licenses/by/4.0/ This content is distributed under the terms of the Creative Commons Attribution 4.0 International license.

Interestingly, the metabolomes of fish in group 1 were clearly separated into two clusters, indicating that the fish mount differential metabolic responses to the *E. tarda* infection at LD_50_. Under this dose, half of the fish died. We hypothesize that the differential metabolomes is associated with differences in the consequences of infection. Indeed, one of these two clusters clustered with the surviving fish (5S) from group 2 (Fig. 2A). Therefore, it is highly possible that the cluster that clustered with 5S was from surviving fish in group 1, which we named 1S, and the other cluster was 1D.

Furthermore, the comparison of the metabolomes of 1S and 1D to 5S identified the metabolites whose abundance were differentially expressed by Mann-Whitney test (Wilcoxon rank sum test) and was displayed as a heat map (Fig. S1D). Z-score varied between −5.82 and 10.64 for 1S to 5S, where 30 metabolites were increased and 26 were decreased (Fig. 2B). Z-score varied between −7.85 and 9.71 of 1D to 5S, where 13 metabolites were increased and 43 were decreased (Fig. 2C). These data suggest that *O. niloticus* adjusts the metabolism to cope with infection that can be predictive of consequences of infection.

### Pathway analysis of differential metabolites upon *E. tarda* infection.

**FIG 3:**
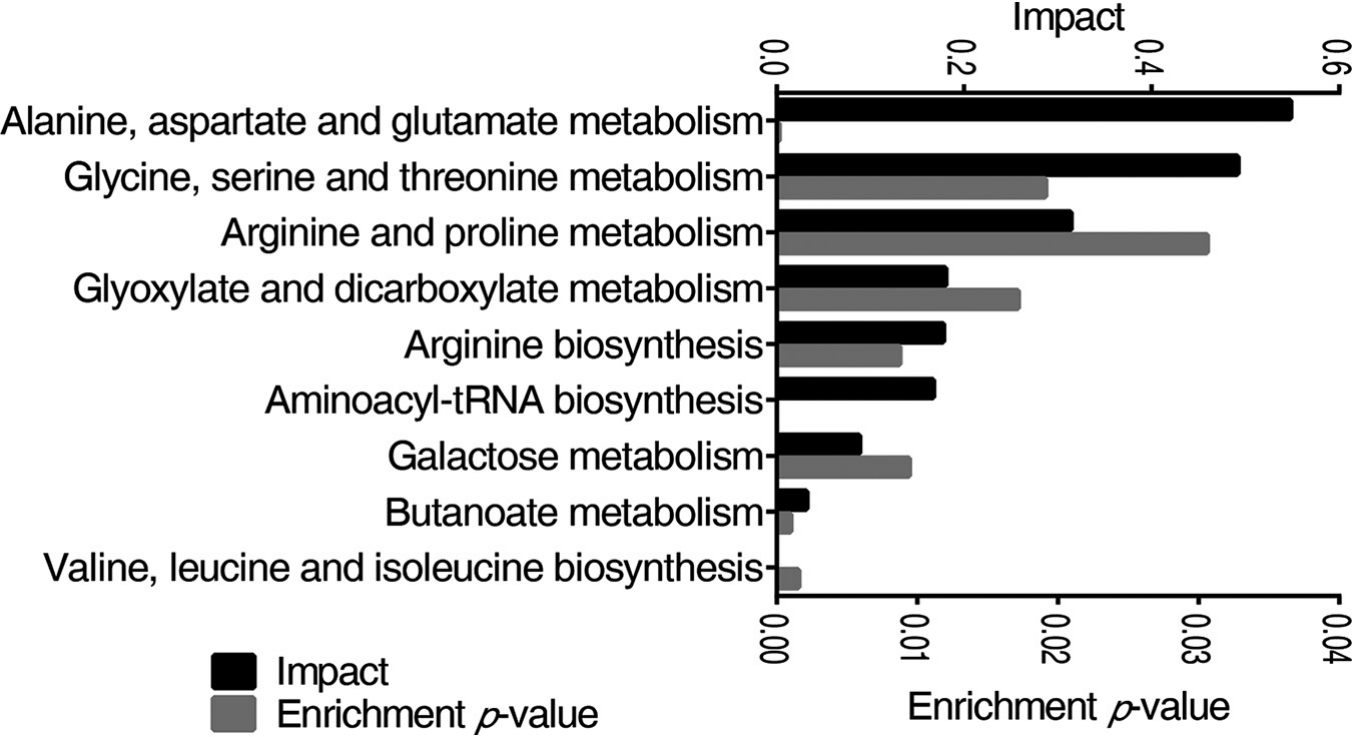
Pathway enrichment analysis of differential metabolites. The metabolites of differential abundance were selected and analyzed in MetaboAnalyst to enrich pathways. Nine pathways that had significant difference (*P < *0.05) were enriched and sorted by their weights (impact).

Pathway analysis provides a comprehensive view of the metabolic flow upon bacterial infection. By MetaboAnalyst 5.0, the metabolites with altered abundance were enriched to nine significant pathways (*P* < 0.05) (Fig. 3), including the metabolism pathway of alanine, aspartate, and glutamate, glycine, serine, and threonine, arginine and proline, glyoxylate and dicarboxylate, arginine biosynthesis, aminoacyl-tRNA biosynthesis, galactose, butanoate, valine, leucine, and isoleucine biosynthesis, which were ordered by their weights (impact). The average levels of differential metabolites in the enriched pathways were shared by the three groups (Table S1A). Despite alanine, aspartate, and glutamate metabolism being the top 2 most impacted pathways, the level of metabolites in other pathways, such as aspartic acid, alanine, and citric acid, were decreased in both 1S and 1D. Therefore, glycine, serine, and threonine metabolism was a potential pathway that promotes fish survival against *E. tarda* infection.

10.1128/mSystems.00426-21.5 TABLE S1 (A) Lists of the metabolites in the enriched pathways of the groups 1D, 1S, and 5. (B) Primers used in this study. Download Table S1, XLSX file, 0.01 MB. Copyright © 2021 Yang et al. 2021 Yang et al. https://creativecommons.org/licenses/by/4.0/ This content is distributed under the terms of the Creative Commons Attribution 4.0 International license.

### Identification of crucial metabolites using multivariate data analysis.

**FIG 4:**
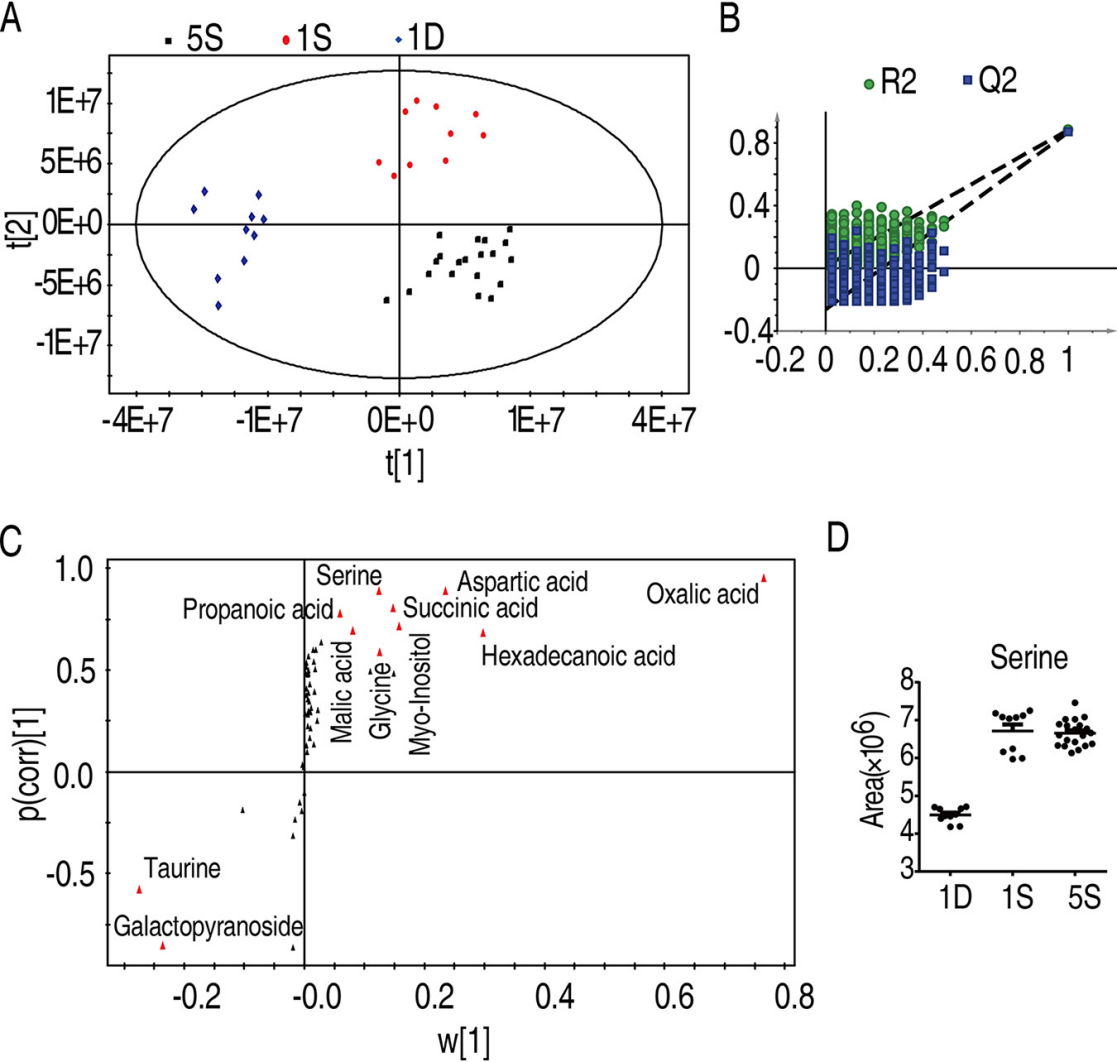
Serine was a crucial biomarker. (A) Principal-component analysis of 1D, 1S, and 5S groups. Each dot represented one technical replicate. (B) The model was monitored using permutation tests. (C) The distribution of differential abundance of metabolites’ weight from PLS-DA to samples. Triangles represent metabolites. Potential biomarkers are highlighted in red. (D) Abundance of serine in 1D, 1S, and 5S. Statistical analysis was performed with Mann-Whitney U test. *, *P < *0.05; **, *P < *0.01. Error bars represented means ± SEM from at least three biological replicates.

Identification of the crucial metabolites that were potent to protect fish against *E. tarda* infection was performed by partial least square discriminant analysis (PLS-DA) (*R*^2^*X* = 0.748, *R*^2^*Y* = 0.855, *Q*^2^ = 0.838). Metabolites from group 1D, 1S, and 5S are clearly separated (Fig. 4A). Component (*t*[1]) distinguishes metabolites of 5S and 1S from that of 1D, and component (*t*[2]) separates metabolites of 5S from the 1S (Fig. 4A). The model was monitored via 999 permutation tests, where the intercepts of *R*^2^ and *Q*^2^ were 0.02 and −0.291, respectively (Fig. 4B), excluding that the model is overfit. The crucial biomarkers that distinguish different groups are highlighted with red in the S-plot (Fig. 4C). The metabolites that differentiated 5S from 1S and 1D include 23 metabolites, namely, inosine, succinic acid, 9,12,15-octadecatrienoic acid, serine, threonine, propanoic acid, malic acid, glucopyranoside, citric acid, mannose, lysine, octadecanoic acid, myoinositol, glutamic acid, hexadecanoic acid, lactic acid, alanine, proline, glycine, aspartic acid, galactopyranoside, oxalic acid, and taurine. The selection of potential metabolites in protecting fish against *E. tarda* infection was based on the abundance of the metabolite being higher in 1S and 5S groups but lower in the 1D group. As such, 17 metabolites fit the criteria, namely, serine, succinic acid, propanoic acid, malic acid, glutamic acid, citric acid, mannose, lysine, octadecanoic acid, lactic acid, myoinositol, glutamic acid, hexadecanoic acid, alanine, proline, glycine, and aspartic acid. All of the identified crucial biomarkers were confirmed with their commercial standards (Data Set S3). Serine is a metabolite that was not only increased in both of the survival groups (1S and 5S) compared to the 1D group but also belonged to one of the most impacted pathways, glycine, serine, and threonine metabolism. Thus, the role of serine in *E. tarda* infection was further explored (Fig. 4D).

10.1128/mSystems.00426-21.8 DATA SET S3 Validation of the crucial biomarkers using their standards. Download Data Set S3, XLSX file, 0.01 MB. Copyright © 2021 Yang et al. 2021 Yang et al. https://creativecommons.org/licenses/by/4.0/ This content is distributed under the terms of the Creative Commons Attribution 4.0 International license.

### The prophylactic effect and therapeutic effect of serine on *O. niloticus* upon *E. tarda* infection.

**FIG 5:**
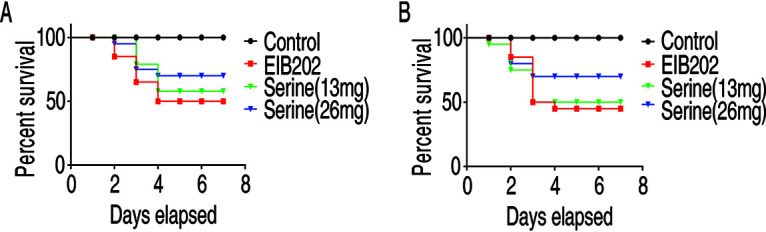
Prophylactic effect and therapeutic effects of serine on *O. niloticus* mortality and *E. tarda* infection. (A) Percent survival of *O. niloticus* challenged with *E. tarda* after serine treatment at the indicated dose. (B) Percent survival of *O. niloticus* challenged with *E. tarda*, which received serine treatment at 1, 4, 10, and 20 h postinfection. The mortality was monitored for a total of 14 days, and only 7 days are shown here. All of the experiments were repeated at least three times.

To examine the prophylactic effects of serine on *E. tarda* infection, *O. niloticus* was daily injected with serine with the indicated doses (13 mg/fish or 26 mg/fish or untreated) for 3 days and then challenged with the LD_50_ of *E. tarda*. The results showed that the survival rate of *O. niloticus* was increased from 50% to 70% in a dose-dependent manner (Fig. 5A). Serine alone had no toxic effects on fish. To test the therapeutic effect of serine, *O. niloticus* was infected with *E. tarda* at LD_50_, followed by serine (13 mg/fish or 26 mg/fish or untreated) injection daily for 3 days. Our results showed that the survival rate of *O. niloticus* was increased about 25% (Fig. 5B). Thus, these data suggested that serine is a promising metabolite to promote host survival against *E. tarda* infection.

### Serine reprograms the metabolome of head kidney-derived leucocytes of *O. niloticus*.

In searching for the mechanism of how serine promotes fish survival, we isolated the monocytes/macrophages from head kidneys after serine treatment for GC-MS analysis. The metabolites were identified by searching against NIST and validated by their RIs (Data Set S4). We have identified a total of 70 metabolites (Data Set S5), where the abundance of 67 metabolites was observed differentially. Z-score varied between −17.50 and 10.85 to control, where 30 metabolites were increased and 26 were decreased by Mann-Whitney test. Z-score varied between −5.82 and 10.64 of 1S to 5S, where the abundance of 30 metabolites was increased and 26 were decreased (Fig. S2A to C). The orthogonal partial least square discriminant analysis (OPLS-DA) (*R*^2^*X* = 0.979, *R*^2^*Y* = 0.999, *Q*^2^ = 0.998) clearly separated the serine-treated group from the control group (Fig. S3A). This model assessed with 999 permutation tests whose *R*^2^ and *Q*^2^ intercepts were 0.003 and −0.305, indicating the reliability of the model (Fig. S3B). The potential biomarkers were identified as shown in Fig. S3C. Abundance of metabolites in the control and serine-treated group is shown in Fig. S4A, and tables list the metabolites of the enriched pathways (Fig. S4B). Of note, serine treatment also increased the intracellular level of serine, as revealed by the metabolomic analysis.

10.1128/mSystems.00426-21.2 FIG S2 Identification of crucial metabolites using multivariate data analysis. (A) Heat map showing relative abundance of metabolites in control and serine groups. (B) Heat map showing relative abundance of different metabolites (Wilcoxon *P* < 0.05) in control and serine groups. Heat map scale (blue to yellow: low to high abundance) is shown at bottom. (C) Z-score plot of differential metabolites based on control. Each point represents one metabolite in one technical repeat and is colored by sample type. Download FIG S2, TIF file, 2.9 MB. Copyright © 2021 Yang et al. 2021 Yang et al. https://creativecommons.org/licenses/by/4.0/ This content is distributed under the terms of the Creative Commons Attribution 4.0 International license.

10.1128/mSystems.00426-21.3 FIG S3 Identification of crucial metabolites using multivariate data analysis. (A) The orthogonal partial least square discriminant analysis of control and serine groups. Each dot represents one technical replicate. (B) The model assessed permutation tests. (C) The potential biomarkers were identified. Download FIG S3, TIF file, 2 MB. Copyright © 2021 Yang et al. 2021 Yang et al. https://creativecommons.org/licenses/by/4.0/ This content is distributed under the terms of the Creative Commons Attribution 4.0 International license.

10.1128/mSystems.00426-21.4 FIG S4 Crucial biomarkers between control and serine groups. (A) Abundance of metabolites in control and serine groups. (B) Tables listing the metabolites of the enriched pathways. Increased metabolites were colored yellow, whereas decreased metabolite was colored blue. Download FIG S4, TIF file, 2.3 MB. Copyright © 2021 Yang et al. 2021 Yang et al. https://creativecommons.org/licenses/by/4.0/ This content is distributed under the terms of the Creative Commons Attribution 4.0 International license.

10.1128/mSystems.00426-21.9 DATA SET S4 Lists of identified metabolites after serine treatment. Download Data Set S4, XLSX file, 0.02 MB. Copyright © 2021 Yang et al. 2021 Yang et al. https://creativecommons.org/licenses/by/4.0/ This content is distributed under the terms of the Creative Commons Attribution 4.0 International license.

10.1128/mSystems.00426-21.10 DATA SET S5 Statistical analysis of metabolomic data before and after serine treatment. Download Data Set S5, XLSX file, 0.05 MB. Copyright © 2021 Yang et al. 2021 Yang et al. https://creativecommons.org/licenses/by/4.0/ This content is distributed under the terms of the Creative Commons Attribution 4.0 International license.

**FIG 6:**
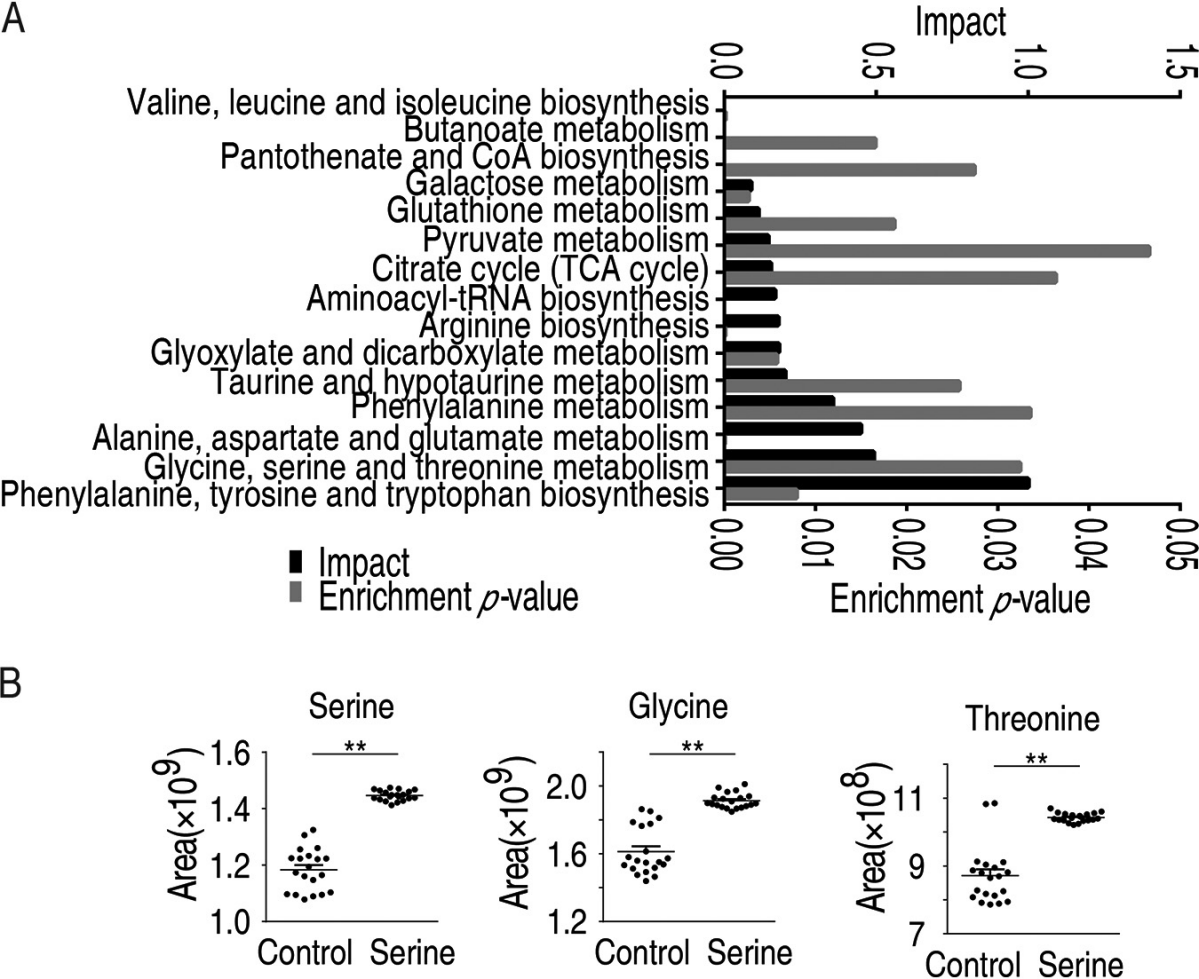
Metabolomic analysis of macrophages/monocytes after serine treatment. (A) Metabolic pathway enrichment analysis of *O. niloticus* treated with serine. (B) Abundance of metabolites in control and serine treatment.

Pathway enrichment analysis of the differential metabolites identified a total of 15 significant pathways (Fig. 6A). The top three pathways included phenylalanine, tyrosine, and tryptophan biosynthesis, glycine, serine, and threonine metabolism, and alanine, aspartate, and glutamate metabolism. As expected, serine treatment increased the glycine, serine, and threonine metabolic pathway as well as the abundance of serine, and glycine, threonine, and cysteine were also increased (Fig. S4B). Interestingly, besides the glycine, serine, and threonine pathway, alanine, aspartate, and glutamate metabolism was also identified (Fig. 3A). This overlap indicated that the two pathways were critical for fish survival upon *E. tarda* infection, and, more importantly, their activations were strongly associated with serine. Since tryptophan was previously shown to increase fish survival, we continued to search for how serine metabolism impacts fish survival. The citric acid cycle was dysregulated, which was consistent with our previous reports that the tricarboxylic acid cycle was not required for combating *E. tarda* infection (18). Interestingly, the glycine, serine, and threonine metabolism were connected to glutathione metabolism, a pathway that was previously not recognized in bacterial infection in *O. niloticus*. Being consistent with the data, the abundance of glycine, threonine, and serine metabolites was increased (Fig. 6B).

### Serine promotes glutathione synthesis.

**FIG 7:**
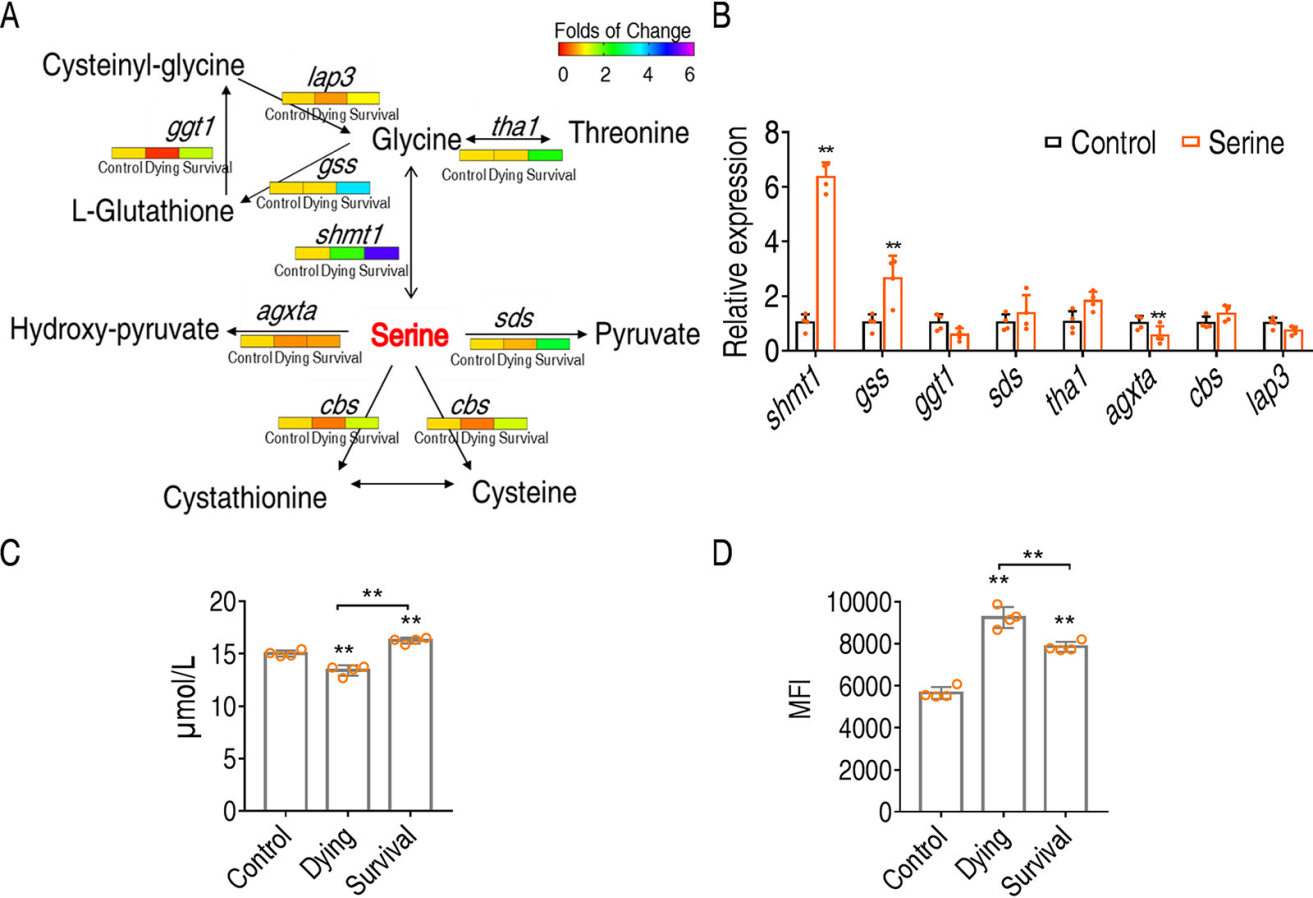
Serine promotes *O. niloticus* survival through glutathione synthesis. (A) Quantification of gene expression in control, dying, and surviving groups. (B) Quantification of gene expression in serine-treated group. (C) Quantification of glutathione in control, dying, and surviving groups. (D) Quantification of ROS in control, dying, and survival groups. Statistical analysis was performed with Mann-Whitney U test. *, *P < *0.05; **, *P < *0.01. Error bars represented means ± SEM from at least three biological replicates. All of the experiments were repeated at least three times.

To understand the action of serine on promoting fish survival against *E. tarda* infection, we quantified the transcription of the genes of serine metabolism during bacterial infection and after serine treatment. According to the metabolic pathway annotation in KEGG, serine can be metabolized to glycine via *shmt1* (serine hydroxymethyltransferase), to hydroxy-pyruvate via *agxta* (serine-pyruvate aminotransferase, mitochondrial), or to pyruvate via *sds* (l-serine dehydratase/l-threonine deaminase). Interestingly, serine metabolism was quite different in the dying group compared to the survival group. Of particular interest was that the genes of serine metabolism were only slightly increased, unchanged, or even decreased in the dying group compared to the control group (Fig. 7A). The expression of those genes was much lower in the dying group than in the survival group. These data suggest that serine metabolism was not properly activated during bacterial infection that would cause fish death. This was also confirmed by serine treatment that upregulates the *shmt1* gene (Fig. 7B). Taken together, these data suggested that serine-to-glycine metabolism plays crucial roles in *O. niloticus* survival.

Next, glycine was metabolized to glutathione via *gss* (glutathione synthetase), to cysteine via *cbs* (cystathionine beta-synthase isoform X1), or to threonine via *tha1* (probable low-specificity l-threonine aldolase 2). The transcriptional expression analysis suggested that serine mainly promoted the biosynthesis of glutathione, as suggested by the upregulated expression of *gss* but downregulated *ggt1* (glutathione hydrolase 1 proenzyme that degrades glutathione) after serine treatment. Thus, the dying group had lower levels of glutathione than the control and survival groups (Fig. 7C). As glutathione is an antioxidant, we found that the dying group has much higher levels of ROS than the survival group (Fig. 7D). These data together suggest that serine promotes fish survival by promoting the metabolic flux to glycine and then glutathione metabolism.

### Serine tunes ROS production during infection.

**FIG 8:**
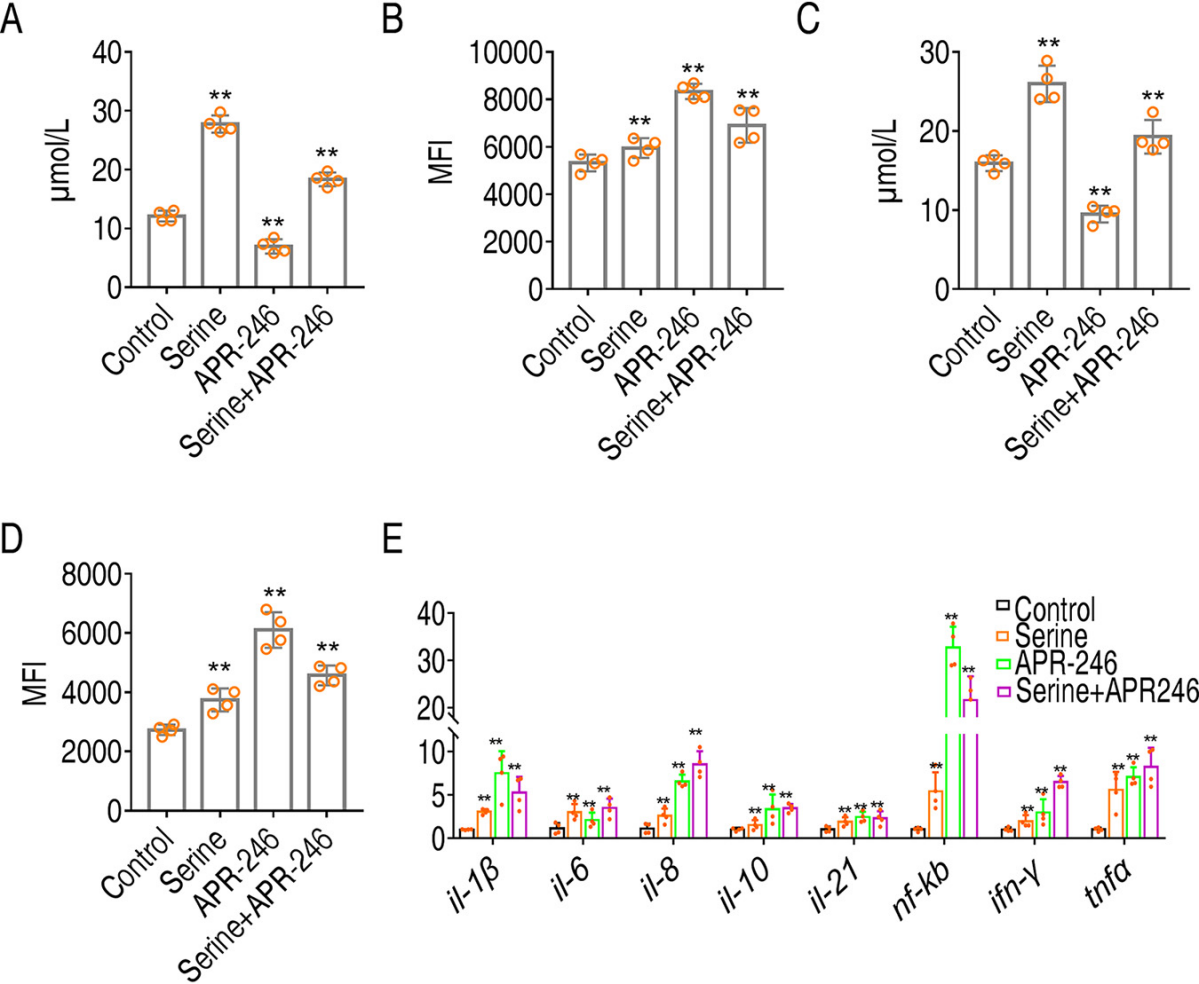
Glutathione negatively regulates ROS production and downregulates immune response. (A) Glutathione when serine served as the prophylactic metabolite in the control group, serine only group, APR-246 only group, and serine plus APR-246 group. (B) ROS when serine served as prophylactic metabolites in control group, serine only group, APR-246 only group, and serine plus APR-246 group. (C) Glutathione when serines served as therapeutic metabolites in control group, survival group, and serine only group. (D) ROS when serines served as therapeutic metabolites in control group, survival group, and serine only group. (E) Quantification of immune gene expression by qRT-PCR. Statistical analysis was performed with Mann-Whitney U test. *, *P < *0.05; **, *P < *0.01. Error bars represented means ± SEM from at least three biological replicates. All of the experiments were repeated at least three times.

Glutathione is known as an antioxidant that scavenges ROS (28), which is critical to induce proinflammatory response, and also accounts for excessive immune response (29). To establish the role of serine-promoted glutathione metabolism in immune response, we measured glutathione and ROS when serine served as prophylactic metabolite and therapeutic metabolite, respectively. *O. niloticus* fish were treated with saline (control group), serine only, APR-246 only (the inhibitor of glutathione synthetase), and serine plus APR-246 once a day for 3 days. *O. niloticus* was intraperitoneally challenged with *E. tarda* EIB202 or saline (control group). The head kidney from tilapia was quantified for glutathione contents and ROS. Serine treatment increased glutathione production (Fig. 8A). However, treatment with APR-246 downregulated glutathione content (Fig. 8A). APR-246 treatment also abolished serine-mediated increases in glutathione and decreases in ROS (Fig. 8A). ROS production was decreased in the presence of ROS but promoted by APR-246 (Fig. 8B). Similar results were obtained with therapeutic effects of serine, which was administered after bacterial challenge (Fig. 8C and D).

**FIG 9:**
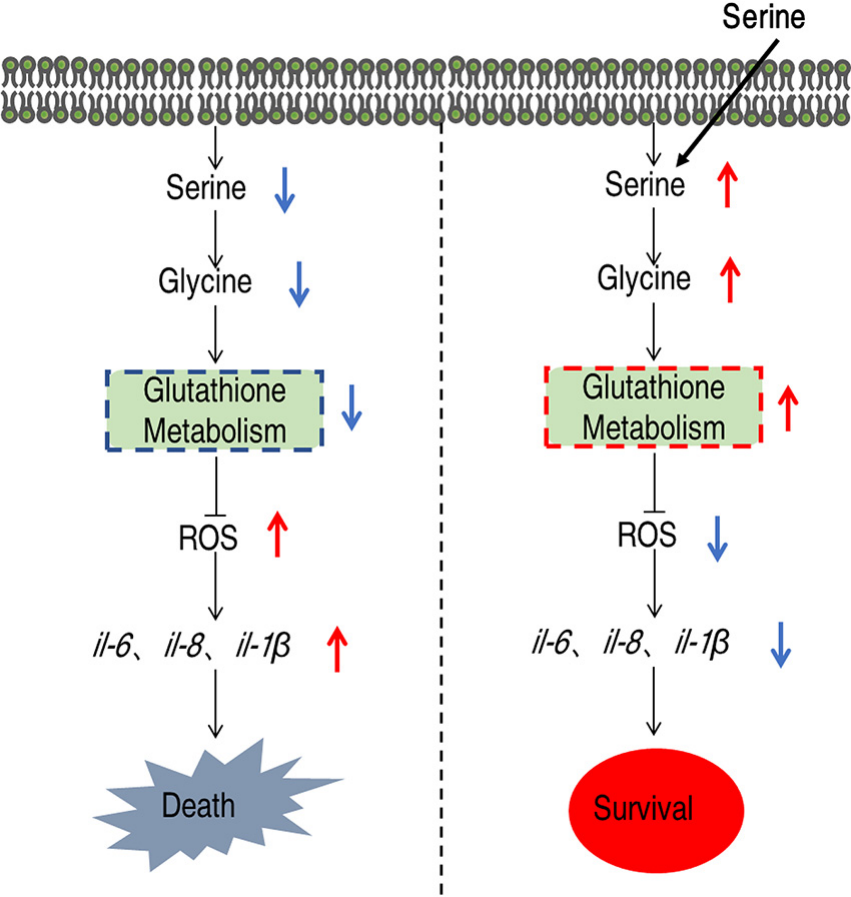
Proposed model. *E. tarda* infection causes tilapia death through virulence factors and septic shock-associated oxidative stress. The oxidative stress could be relieved by serine, which fluxes into the glutathione metabolism to reduce ROS production. Glutathione antagonized the ROS, thereby protecting host death from overactive immune responses like ROS.

The relationship between serine, glutathione, and immune response was established by quantifying the expression of immune genes. As shown in Fig. 8E, APR246 treatment augmented the gene expression level of all the tested genes compared to the serine-treated group. More importantly, APR246 together with serine induced higher levels of gene expression than serine (Fig. 8E). These data suggest that serine-promoted glutathione production is the mechanism by which serine tunes immune responses to avoid overreactive responses (Fig. 9).

## DISCUSSION

### Modulating immunity-based approach to fight bacterial infection is urgently needed.

Bacterial infection is still a risk factor for human health and poultry, taking large numbers of lives per year and causing huge economic loss (30). Due to widespread antibiotic-resistant bacteria, antibiotics are used with caution, and a backup strategy to deal with the infection is urgently needed. Boosting immune response is one of the most enticing strategies, since it is built upon one’s own immune system and unlikely to produce antibiotic-resistant bacteria (31). Great progress has been made in the past in elucidating host-pathogen interactions, and the key molecules in mediating immune response toward bacterial infection have been identified (32). It is now well established that pathogen is not the only factor that causes host death; overactive immune response also contributes significantly to death. Therefore, tuning immune responses to certain extents is crucial for elimination bacterial infection without damaging the host itself, but the preventive measure for the control of excessive immune responses is still lacking.

### Reprogramming metabolomics is potent in identifying metabolites to regulate host immune response upon bacterial infection.

In the present study, we present reprogramming metabolomics as an approach to identify potential metabolic modulators of excessive immune response. First, we showed that *O. niloticus* mounted a metabolic shift upon *E. tarda* infection that is predictive of the consequence of infection. We have previously shown that dying fish and surviving fish presented different metabolomic profiles, and the protective metabolite(s) can be identified (18). It is to our surprise that the different metabolomic profiles can be distinguished as early as 6 h postinfection. We can expect that metabolic signature is a predictive factor for assessing the consequences of infection, and we can also develop control measures to prevent advancement of infectious diseases at the onset of infection. This is consistent with the idea that metabolism is the guiding force for immunity, which highlights the roles of the metabolite(s) or metabolic pathways in providing energy or in regulating the response (33). What we found here is that metabolome as a whole functions in regulating immune response. Actually, this idea has been well proved in our research on antibiotic-resistant bacteria that the metabolomic profile can be used to distinguish antibiotic-resistant and -sensitive bacteria and serum-resistant and -sensitive bacteria. The crucial biomarker can be used to reprogram the metabolome to revert the resistance to antibiotics or serum (34). Therefore, metabolome reprogramming can be extensively explored for the combat of infection.

### ROS is critical for host survival upon bacterial infection.

Serine is a crucial metabolite that emerged at 6 h postinfection to promote fish survival by downregulating ROS. Our study revealed that ROS production occurred early upon infection, and tuning ROS level is critical for host survival and can be done early in the infection. Actually, ROS acts as a second messenger by activating key pathways in defending against pathogens, exemplified by its role in macrophage (35). For example, ROS production is critical for the activation of NLRP3, which is crucial for inflammasome activation, and the activation of caspase-1 for interleukin-1β (IL-1β) maturation (36, 37). ROS scavenger blocks inflammasome activation (38). In contrast, constant high levels of ROS cause hyperactivation of immune response that injure the tissues (39). Thus, balancing ROS production is crucial for maintaining homeostasis.

### Serine plays important roles in host survival.

As a nonessential amino acid, serine is required for cell proliferation (40). The metabolism of serine involves one-carbon metabolism. The role of serine on cancer cell and T-cell proliferation is well recognized to provide substrates for the synthesis of nucleotides, NADPH, and glutathione (41, 42). It was recently shown that serine supports lipopolysaccharide-induced IL-1β production in macrophages (43). However, how serine engages in host-pathogen interaction, and whether it contributes to host survival, was largely unexplored. Our data suggest that serine metabolism plays an early role in mediating pathogen-induced immune response. More importantly, enhancement of serine metabolism can decrease the level of overactive immune response to promote cell survival.

### Serine is a potential metabolite to be used in aquaculture.

The dose of serine we used in this study is safe. Serine was a supplement in daily life years ago and is found at high levels in food such as soybeans, peanuts, and almonds (44). The benefits of daily intake of serine include improvement of brain function in patients with dementia, stress relief, fighting fibromyalgia, improvement of sleep, and therapy for cancer (45, 46). A study showed that treatment with l-serine at a dose of 400 mg/kg of body weight/day for a 52-week duration did not cause observable side effects (47). Therefore, the dose at 26 mg/per fish we used in this study is within a safe range.

### Limitations of this study.

There are three limitations of this study. First, this study only focuses on the metabolic differences between the surviving and dying fish but not the predictors that may account for excessive immune response or even systemic immune challenge. This is because we only used several selected immune genes for this study, which were only investigated by quantitative real-time PCR (qRT-PCR), and were difficult to correlate with the metabolomic data in a multivariate study. The second limitation is that we used fish as a whole to investigate the correlation between metabolite and immune response. However, we can hardly establish the relationship between individual metabolite and individual cytokine expression due to the complex metabolic networks and different tissues in driving certain immune responses, especially at the organismal level. Further study can be performed on the cellular level. Moreover, we only focus our study on how the major pathways were affected and its corresponding contribution to the host-pathogen interaction. However, how these pathways were connected to each other was not explored. This information may provide a much more comprehensive view of disease advancement.

In conclusion, we identify serine as a crucial biomarker that distinguishes the surviving fish and dead fish that were infected by bacteria. Serine is a potential metabolite to be used in aquaculture to reduce overactive immune response during bacterial infection.

## MATERIALS AND METHODS

### Ethics statement.

This study was conducted in accordance with the recommendations in the *Guide for the Care and Use of Laboratory Animals* of the National Institutes of Health (48). Animals were maintained according to standard protocols. All experiments were approved by the Institutional Animal Care and Use Committee of Sun Yat-sen University (approval no. SYSU-IACUC-2020-B126716).

### Fish and rearing conditions.

Nile tilapia (*Oreochromis niloticus*), hatched from the same batch of fertilized eggs and collected at 65 ± 5 days postfertilization with a weight of 100 ± 2 g, were obtained from Guangdong Tilapia Breeding Farm (Guangzhou, China) with equal numbers of males and females. *O. niloticus* was maintained in 25-liter tanks equipped with closed recirculating aquaculture systems, and the maintenance physicochemical parameters were the following: water temperature, 28°C; dissolved oxygen, 6 to 7 mg/liter; carbon dioxide content, <10 mg/liter; pH value, 7.0 to 7.5; nitrogen content, 1 to 2 mg/liter; and nitrite content, 0.1 to 0.3 mg/liter. These animals were acclimated under this condition for 2 weeks before the experiment and were fed twice daily with commercial fish feed on a 12-h/12-h rhythm of light and dark photoperiods. The tank was cleaned twice a day by siphoning up the food debris and feces.

### Bacterial strains and infection dose.

*E. tarda* EIB202 (CCTCC no. M208068) was a generous gift from X. H. Zhang, Ocean University of China. A single colony of *E. tarda* EIB202 was picked up from a tryptic soy broth (TSB) plate and propagated overnight in 100 ml TSB medium at 200 rpm at 30°C. The culture was diluted 1:100 into fresh TSB medium until the optical density at 600 nm was 1.0. Bacteria were harvested by centrifugation at 6,000 × *g* for 5 min. The cells were washed three times with saline solution and finally resuspended in saline solution for infection.

To determine the lethal dose, *O. niloticus* fish were intraperitoneally infected with three different doses of bacteria, including 0.8 × 10^8^, 1.6 × 10^8^, and 3.2 × 10^8^ CFU/fish. There were 20 *O. niloticus* fish at each dose. The mortality was monitored twice a day for a total of 14 days. Under 1.6 × 10^8^ CFU/fish dose, dying fish and surviving fish head kidneys at 48 h postinfection were collected to quantify the expression of immune-related genes.

### Isolation of monocytes/macrophages from head kidney and sample preparation for GC-MS.

To identify metabolic signature associated with *O. niloticus* upon *E. tarda* infection, monocytes/macrophages isolated from *O. niloticus* challenged with the LD_50_ of *E. tarda* were analyzed. Forty *O. niloticus* fish were randomly divided into two groups with 20 animals in each group. As shown in Fig. S1A in the supplemental material, animals in one group were sacrificed at 6 h postinfection, and animals in the other group were considered surviving fish 5 days postinfection, after which no more fish would die. Isolation of monocytes/macrophages was performed as previously described (49). Briefly, head kidneys were aseptically removed and pressed through an 80-μm sterile steel mesh to exclude unhomogenized tissues. The filtrates were then resuspended in L-15 medium (Gibco, USA) supplemented with 10% fetal bovine serum (FBS) (Gibco, USA) and 1% penicillin-streptomycin (Sigma, USA). The cell suspensions were layered onto a 54%/31% discontinuous Percoll (Sigma, USA) density gradient and centrifuged at 400 × *g* at 4°C for 40 min. The cells were washed three times with serum-free medium at 400 × *g* and used immediately for GS-MS preparation.

The total metabolites were extracted from cells as previously described (50). Briefly, the cells were quenched with cold methanol containing 10 μl of 0.1 mg/ml ribitol (Sigma-Aldrich, USA), which served as an analytical internal standard. Cells were then lysed by sonication for 10 min and centrifuged for another 10 min at 12,000 rpm at 4°C. The supernatant was dried in a rotary vacuum centrifuge device (LABCONCO).

The dried extracts were derivatized as previously described (51); 80 μl of 20 mg/ml^−1^ methoxyamine hydrochloride (Sigma-Aldrich, USA) was added to the dried samples, mixed, and incubated at 37°C for 90 min; 80 μl N-methyl-N-(trimethylsilyl) trifluoroacetamide (MSTFA; Sigma-Aldrich) was added, followed by incubation at 37°C for another 30 min. The derivatized samples were centrifuged and supernatants were collected. The sample (1 μl) was injected into a 30-m by 250-μm (inner diameter) by 0.25-μm DB-5MS column using splitless injection via an Agilent autoinjector. The initial temperature of the GC oven was held at 85°C for 5 min, followed by an increase to 270°C at a rate of 15°C min^−1^ and then maintained for 5 min. Helium was used as the carrier gas at a constant flow rate of 1 ml min^−1^. Electron impact ionization (70 eV) at full scan mode (*m/z* 50 to 600) at a rate of 20 scans s^−1^ was used.

Retention index (RI) was performed with alkane standards (alkane mix 34, CDAA-M-690038-HD-1ml; ANPEL Laboratory Technologies [Shanghai] Inc., Shanghai, China), which includes *n-*octane (CAS: 111-65-9), *n-*decane (CAS: 124-18-5), *n-*dodecane (CAS: 112-40-3), *n-*tetradecane (CAS: 629-59-4), *n-*hexadecane (CAS: 544-76-3), *n-*octadecane (CAS: 593-45-3), *n-*eicosane (CAS: 112-95-8), *n-*docosane (CAS: 629-97-0), *n-*tetracosane (CAS: 646-31-1), *n-*hexacosane (CAS: 630-01-3), *n-*octacosane (CAS: 630-02-4), *n-*triacontane (CAS: 638-68-6), *n-*dotriacontane (CAS: 544-85-4, *n-*tetratriacontane (CAS: 14167-59-0), *n-*hexatriacontane (CAS: 630-06-8), *n-*octatriacontane (CAS: 7194-85-6, *n-*tetracontane (CAS: 4181-95-7), *n-*heptane (CAS: 142-82-5), *n-*nonane (CAS: 111-84-2), *n-*undecane (CAS: 1120-21-4), *n-*tridecane (CAS: 629-50-5), *n-*pentadecane (CAS: 629-62-9), *n-*heptadecane (CAS: 629-78-7), *n-*nonadecane (CAS: 629-92-5), *n-*heneicosane (CAS: 629-94-7), *n-*tricosane (CAS: 638-67-5), *n-*pentacosane (cas: 629-99-2), *n-*heptacosane (CAS: 593-49-7), *n-*nonacosane (CAS: 630-03-5), *n-*hentriacontane (CAS: 630-04-6), *n-*tritriacontane (CAS: 630-05-7), *n-*pentatriacontane (CAS: 630-07-9), *n-*heptatriacontane (CAS: 7194-84-5), *n-*nonatriacontane (CAS: 7194-86-7). The RIs of the metabolites were calculated by RI*_x_* = 100 × *z* + 100 × (RT*_x_* − RT*_z_*)/(RT*_z_*_+1_ − RT*_z_*), where RI*_x_* is the retention index of metabolite *x* and RT*_x_*, RT*_z_*, and RT*_z_*_+1_ were retention times of metabolite *x*, alkane *z*, and *z* + 1, respectively. Here, *z* and *z* + 1 represent the carbon atom’s number of alkanes.

### Metabolite identification and data processing.

Date processing was performed as previously described (52). GC-MS chromatograms were analyzed using ChemiStation (ChemiStation C.01.01), and each peak was integrated individually. The deconvolution and calibration of raw data mass spectra were performed with AMDIS (Agilent OpenLAB CDS). Metabolites were identified by retrieving their mass spectra in the NIST 2011 library (National Institute of Standards and Technology, USA) and GMD 2011 (Golm Metabolome Database, Germany) according to the following criteria: match value of ≥750, reverse match value of ≥800, and a probability of ≥60% (53). Peaks with a signal-to-noise ratio greater than 30 were reserved to avoid false positives (54). The raw data of mixed alkanes are automatically imported into AMDIS, and the retention index of each substance is generated by AMDIS. The combined normalization processing consisted of the following options (55): quantile normalization row-wise procedures and center scaling (the variable is centered but not scaled [ws = 1]). The zeroes or missing values were assessed by the singular value decomposition (SVD) method to impute the missing values (56). The data matrix was used for the subsequent multivariate statistical analysis. For the spectra that were not identified as described above, ion fragments were manually extracted from the extracted ion chromatography (EIC) and searched against GOLM (http://gmd.mpimp-golm.mpg.de/) (57) and MassBank (https://massbank.eu/MassBank/Search) (58) for metabolite retrieval and identification, and RI was used to confirm identity.

Metabolomic standards initiative (MSI) level of the reported metabolites was determined as described previously (59). In level 1, identified metabolites are confirmed with authentic compound standards with at least two ortholog parameters; in level 2, identified metabolites are similar to the spectrum in public or commercial databases; in level 3, metabolites were identified to a class or similar known compounds. Level 4 is unidentified metabolites. Two methodologies were adopted to minimize batch variation. First, the internal standard ribitol was used to monitor the quality control from batch to batch. Second, the instrument was calibrated with GC-MS tuning standard PFTBA (05971-60571l; Agilent Technologies, USA) after instrument maintenance.

### Statistical analysis, multivariate data analysis, and pathway enrichment analysis.

Statistical analysis was performed with SPSS 13.0. The difference among metabolites was determined with Mann-Whitney U test and Kruskal-Wallis test as described previously, where a *P* value of <0.05 was considered statistically significant (60).

Z-score was used to analyze normalized area of differential metabolites. Z-score analysis scaled each metabolite according to a reference distribution (18). The mean and standard deviation from each metabolite were determined in these samples. *O. niloticus* was incubated with *E. tarda* EIB202. The first day of survival (1S) and the first day of dying (1D) were centered by the 5 days of survival (5S) mean and scaled by the preinfection standard deviation. PLS-DA and orthologous PLS-DA (OPLS-DA) were performed by SIMCA 14.0 (Umetrics, Umeå, Sweden) to identify patterns associated with infection and minimize influence of the interindividual variation. To exclude the possible overfit of the model, a 10-fold cross-validation and permutation test (999 times) were adopted. The overfit was monitored by the intercept on the *y* axis, wherein a value of *Q*^2^*Y* of less than 0.05 indicates a good model (61). In addition, the pattern recognition model was assessed based on the values of *R*^2^ and *Q*^2^, both of which together indicate the quality of the model. Therefore, the model is robust when *R*^2^ > 0.5 and *Q*^2^ > 0.4 (62). Data were log transformed prior to data import. Center scaling was selected before fitting. All variables were mean centered and scaled to standard deviation of each metabolite.

Enrichment of significant pathways was conducted with MetaboAnalyst 5.0 (63). The input data were limited to metabolites with significant differential. The −log(*p*) value and a value reflecting the impact of each metabolic pathway were calculated using a hypergeometric test, and the metabolic pathways with a *P* value of <0.05 were considered significant. Comparative metabolic pathway analysis between the two groups was performed with iPath 3.0 (https://pathways.embl.de/).

### Prophylactic effect and therapeutic effect of serine.

For prophylactic effect of serine, 80 *O. niloticus* fish were randomly divided into four groups with equal numbers in each group. Fish in two of these groups were treated with saline, and those in another two groups were treated with either 13 mg or 26 mg serine per fish once a day for 3 days. *O. niloticus* was challenged with EIB202 at 1.6 × 10^8^ CFU/fish, except for those in the one group treated with saline. Cumulative death was monitored twice a day for a total of 14 days.

For the study of therapeutic effect of serine, 80 *O. niloticus* fish were randomly divided into three groups with 20 fish in each group. Three of the groups received challenge with EIB202 at 1.6 × 10^8^ CFU/fish, among which two of the groups received serine treatment either with 13 mg/fish or 26 mg/fish at 1, 4, 10, and 20 h postinfection. The group that received the same volume of saline was the negative control. Cumulative death was monitored twice a day for a total of 14 days.

### qRT-PCR.

qRT-PCR was carried out as described previously (64, 65). Total RNA of each sample was isolated with TRIzol (Invitrogen, USA). The RNA was then quantified spectrophotometrically. qRT-PCR was carried out on 1 μg of total RNA by using an EvoM-MLV RT kit with gDNA clean for qPCR (AG11705; Accurate Biotechnology) according to the manufacturer’s instructions. The cDNAs were synthesized with a reverse transcription kit and used as the template for qRT-PCR. qRT-PCR was performed in 384-well plates with a total volume of 10 μl containing 5 μl 2× SYBR green premix pro *Taq* HS qPCR kit (AG11701; Accurate Biotechnology), 2.6 μl H_2_O, 2 μl cDNA template, and 0.2 μl each of forward and reverse primers (10 μM). All samples were tested in biological triplicate and run on a CFX384 Touch (Bio-Rad, USA) according to the manufacturer’s instructions. The cycling parameters were 95°C for 30 s to activate the polymerase; 40 cycles of 95°C for 10 s; and 56°C for 30 s. Fluorescence measurements were performed at 72°C for 1 s during each cycle. Cycling was terminated at 95°C with a calefactive velocity of 5°C/s to obtain a melting curve. The expression of samples was normalized by *β-actin*, *gapdh*, and *ef1α* using the 2^−ΔΔ^*^CT^* method (66). All the primers are listed in Table S1B.

### Quantification of reactive oxygen species.

The production of ROS was quantified by DCFH-DA, with minor modifications (14). The head kidneys from tilapia were pooled and homogenized in ice-cold phosphate-buffered saline (pH 7.4) at a ratio of 1:15 (wt/vol). The homogenates were centrifuged to remove debris, and the supernatant was collected and mixed with 25 μmol 2′,7′-dichlorofluorescin diacetate solution (Sigma, USA) for 30 min at 37°C in the dark. Fluorescence of the samples was monitored at an excitation wavelength of 490 nm and an emission wavelength of 515 nm by a microplate reader (Varioskan LUX; Thermo Scientific). The results were analyzed with Mann-Whitney U test. *, *P < *0.05; **, *P < *0.01.

### Determination of glutathione.

Glutathione content was assessed using a commercial assay kit (Boshen Biotechnology Co., Ltd., Nanjing, China). The head kidneys from tilapia were pooled and homogenized in ice-cold phosphate-buffered saline (pH 7.4) at a ratio of 1:15 (wt/vol). For glutathione, the supernatant was added to encapsulated micropores precoated with captured antibodies, and then 100 μl of horseradish peroxidase-conjugated reagent was added to each well, which was then covered with an adhesive strip and incubated for 60 min at 37°C. Solution in each well was then aspirated, followed by three washes. After the last wash, the residual wash solution was removed by aspirating or decanting. Chromogen solution A and chromogen solution B were added to each well, followed by 15 min of incubation in the dark at 37°C. A volume of 50 μl stop solution was then added to each well. The optical density at 450 nm in each well was recorded with a microtiter plate reader.

### Data availability.

All of the metabolomic raw data were deposited to MetaboLights (http://www.ebi.ac.uk/metabolights/) (67). The unique identifier is MTBLS2974, which can be found through the link www.ebi.ac.uk/metabolights/MTBLS2974.
