# Supplementary figures and images for: Serine Metabolism Tunes Immune Responses To Promote Oreochromis niloticus Survival upon Edwardsiella tarda Infection
Source: mSystems. 2021 Aug 24;6(4):e00426-21. doi: 10.1128/mSystems.00426-21 (PMC8407201; doi:10.1128/mSystems.00426-21)

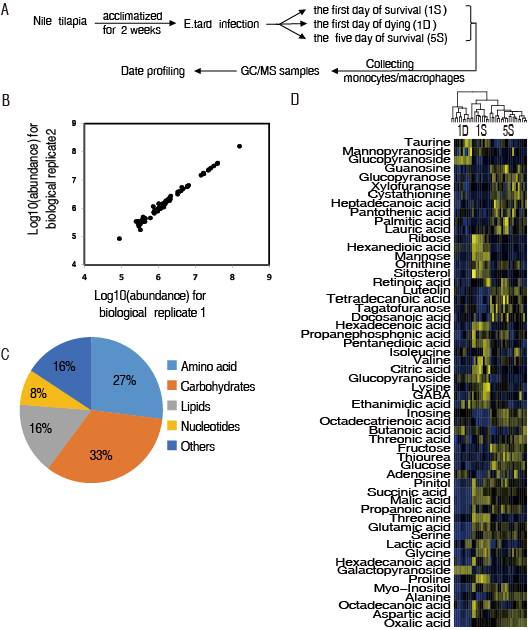

Supplement: FIG S1 [file msystems.00426-21-sf001.tif]

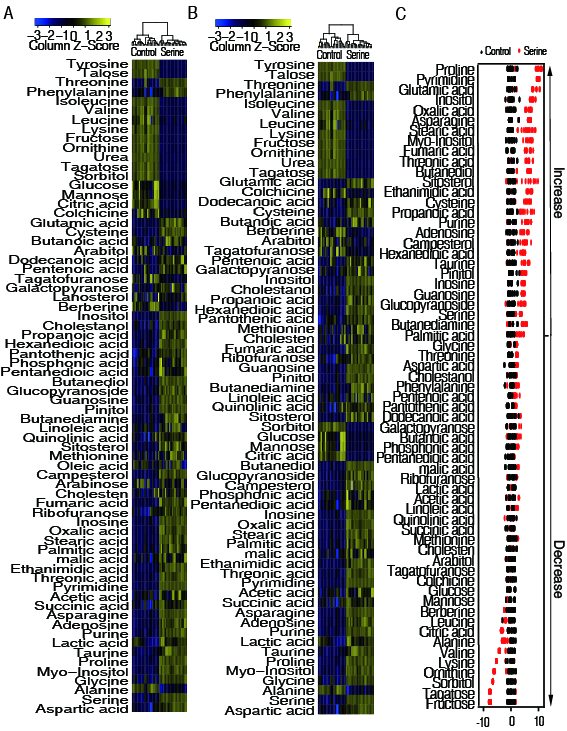

Supplement: FIG S2 [file msystems.00426-21-sf002.tif]

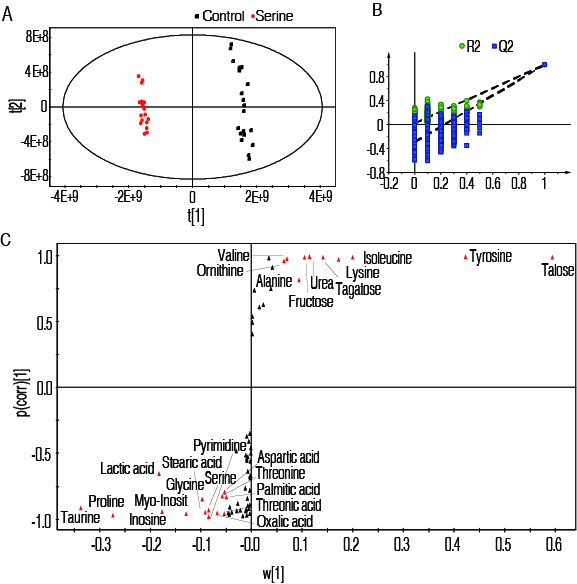

Supplement: FIG S3 [file msystems.00426-21-sf003.tif]

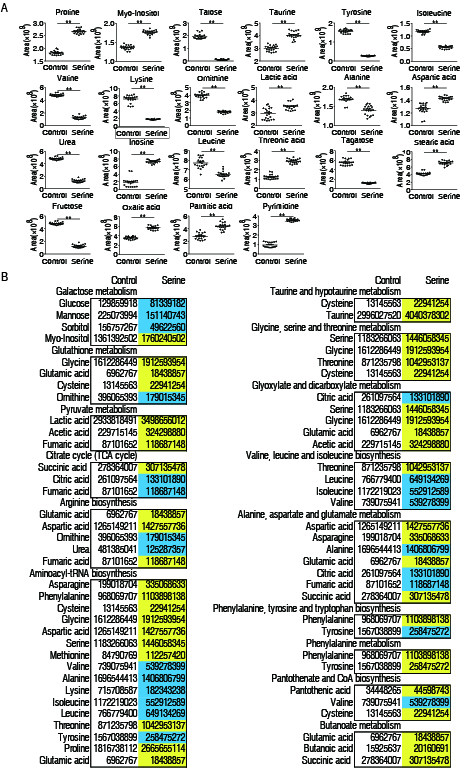

Supplement: FIG S4 [file msystems.00426-21-sf004.tif]
